# Concentration of Polyphenolic Antioxidants in Apple Juice and Extract Using Ultrafiltration

**DOI:** 10.3390/membranes12111032

**Published:** 2022-10-23

**Authors:** Mariya Dushkova, Kiril Mihalev, Angel Dinchev, Kiril Vasilev, Diyan Georgiev, Margarita Terziyska

**Affiliations:** 1 Department of Food Preservation and Refrigeration Technology, University of Food Technologies, 26 Maritza Blvd., 4002 Plovdiv, Bulgaria; 2 Research Institute of Mountain Stockbreeding and Agriculture, 281 Vasil Levski Str., 5600 Troyan, Bulgaria

**Keywords:** ultrafiltration, apple juice, apple extract, polyphenolic

## Abstract

The aim of the present work was to study the potential of ultrafiltration with three polyacrylonitrile membranes (1, 10, and 25 kDa) to concentrate polyphenolic antioxidants in apple juice and extract. The permeate flux, total polyphenols, polyphenolic profile, phenolic acid content, and total antioxidant capacity were determined using the FRAP and DPPH tests, the content of water-soluble proteins during ultrafiltration was established, and the concentration factors and rejections were determined. The permeate flux decreased by increasing the volume reduction ratio and decreasing the molecular weight cut-off of the membranes. The concentration factor and rejection of polyphenolics increased with the increase in the volume reduction ratio (VRR) for all membranes and both liquids. The concentration and rejection effectiveness of the 1 kDa membrane was higher than those observed for 10 and 25 kDa during the ultrafiltration of the apple extract, while these values were comparable for 1 and 10 kDa during the ultrafiltration of the apple juice. The concentration factors and rejections of total polyphenols were higher in the extract than in the juice. Chlorogenic acid was the main compound in the polyphenol profile of apple juice. The total content of phenolic acids, determined by using HPLC, increased by 15–20% as a result of the membrane concentration, but the separation process did not significantly change the ratio between the individual compounds.

## 1. Introduction

The plants are rich in flavonoids and polyphenolics and have high antioxidant activity. They are widely used in medicine because they have many healthy effects, including anti-inflammatory and antirheumatic, antiallergic, anticancer, and antiviral properties [1,2,3].

Nowadays, the determination of the concentration of polyphenols by using membrane technologies is important from a scientific and industrial point of view because the low temperatures of treatment allow researchers to avoid the thermal degradation of these compounds. Usually, nanofiltration is used for the fractionation and concentration of flavonoids, anthocyanins, carotenoids, sugars, and phenolic compounds from fruit and vegetable matrices [4], while ultrafiltration is mainly used for the purification of juices [5,6]. Moreover, fractionation, purification, and concentration of polyphenols are of healthy and economic interest to the food industry [7]. The traditional methods for concentrating biologically active compounds, extracted from natural products, apply simple steam and vacuum distillation and thus use high temperature and energy consumption [8]. The first is inapplicable for heat-sensitive products. These methods may induce a loss of compounds of low molecular weight, which can be removed together with the solvent during evaporation [9]. The traditional concentration methods can be replaced by membrane processes because they are carried out without phase transition and at a relatively low temperature, which preserves the native characteristics of the separated components [10,11]. Other advantages of the membrane processes are environmental friendliness [12], lower power consumption [13], increased yield [14,15], higher quality of the final product [16,17], reduction in production costs [18], and high selectivity [19].

Membrane technologies are applied in many branches of food production, including the fruit juice industry. Many researchers have directed their efforts to study the effects of important factors such as membrane filtration, interpretation, comparison of results, and the practical use of membrane processes and proved that the juices produced via membrane filtration have an excellent quality according to their physicochemical and sensory characteristics [20].

The hydrophobic nature and molecular weight cut-off (MWCO) of the membrane may affect the separation and concentration of valuable compounds [8,21,22]. The type of the liquid and the level of concentration also have a significant influence on the main characteristics of the process (permeate flux, concentration factor, and rejection) [20].

The aim of the present work was to study the potential of ultrafiltration with three different membranes to concentrate polyphenolic antioxidants in apple juice and extract.

## 2. Materials and Methods

### 2.1. Materials

#### 2.1.1. Chemicals

The following reagents and standards were used: DPPH [2,2-diphenyl-1-picrylhydrazyl], TPTZ [2,4,6-tripyridyl-s-triazine], and Trolox [(±)-6-hydroxy-2,5,7,8-tetramethylchromane-2-carboxylic acid] (Sigma-Aldrich, Steinheim, Germany); Folin–Ciocalteu reagent (FC-reagent) (Merck, Darmstadt, Germany); gallic acid monohydrate, chlorogenic acid, caffeic acid, and *p-*cumacic acid (Fluka, Buchs, Switzerland).

All other reagents and solvents had analytical or HPLC grades.

#### 2.1.2. Plant Materials

##### Apple Extract

The VitaRed™ apple extract (70% polyphenols) was provided by Future Ceuticals (Momence, IL, USA). The model solution (0.1%, *w*/*v*) of the apple extract was prepared with distilled water, acidified (pH 3.0) with 1 M HCl to be close to the pH value of the apple juice from the studied concentrate (pH 3.34), via treatment (20 min) in an ultrasonic bath (USC200T, VWR International bvba/sprl, Leuven, Belgium).

##### Apple Juice from Concentrate

Depectinized cloudy apple concentrate (≥70 Brix) was provided by Krichimfrukt Ltd. (Krichim, Bulgaria). The concentrate was diluted with distilled water up to a soluble solid content of 11.2 Brix. To achieve the necessary clarity (transmittance, T ≥ 90%), the juice was sequentially filtered through filter paper and paper filter Filtrak 390 (VEB Spezialpapierfabrik Niederschlag, Niederschlag/Erzgebirge, Germany).

### 2.2. Methods

#### 2.2.1. Membrane Equipment

**Figure 1:**
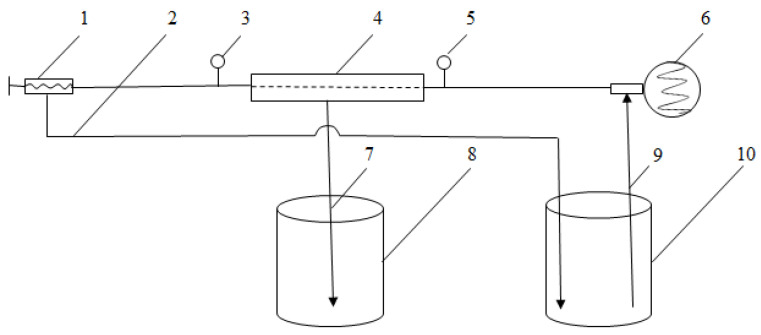
Scheme of laboratory membrane equipment: 1—pressure regulator; 2—pipeline for retentate; 3—manometer (0–1 MPa); 4—replaceable plate and frame membrane module; 5—manometer (0–1 MPa); 6—3-frame piston pump; 7—pipeline for permeate; 8—tank for permeate; 9—pipeline for feed solution/retentate; 10—tank for feed solution/retentate.

Membrane filtration was carried out using laboratory equipment with a replaceable plate and a frame membrane module with a membrane area of 1250 cm^2^, shown in Figure 1. Polyacrylonitrile asymmetric membranes UF1-PAN, UF10-PAN, and UF25-PAN with molecular weight cut-offs (MWCO) of 1 kDa (average pore size 1 nm), 10 kDa (average pore size 1.5 nm), and 25 kDa (average pore size 2 nm), respectively, were used. The membranes were produced at the University of “Prof. Dr. Asen Zlatarov”, Bulgaria. The volume of the feed solution (*V*_F_) was 6 L for each ultrafiltration experiment. The experiments were conducted under the following working conditions: operating pressure 0.4 MPa; temperature 20 °C; feed flow rate 330 dm^3^/h; volume reduction ratio 2, 4, and 6. The membranes were cleaned with NaOH 0.5%, a temperature of 50 °C, pressure of 0.2 MPa, circulation time of 30 min, and final rinsing with distilled water.

#### 2.2.2. Calculation of Main Characteristics of Ultrafiltration Process

The permeate flux (J, L/(m^2^h)) was calculated according to the following formula:

(1) $$J=\frac{V}{A*t}$$

where *V* is the volume of collected permeate, L; *A* is the membrane area, m^2^; *t* is the time, h.

The volume reduction ratio (VRR) was calculated as follows:

(2) $$VRR=\frac{V_{F}}{V_{R}}$$

where *V_F_* is the volume of the feed solution, dm^3^; *V_R_* is the volume of the retentate obtained during ultrafiltration, dm^3^.

The rejection (R, %) was calculated using the following formula:

(3) $$R=\left(1-\frac{C_{p}}{C_{R}}\right)*100,\;\%$$

where *C_P_* is the concentration of the component present in the permeate, %; *C_R_* is the concentration of the component present in the retentate, %.

For calculating the concentration factor (CF), the following equation was used:

(4) $$CF=\frac{C_{R}}{C_{O}}$$

where *C_R_* is the concentration of the component present in the retentate, %; *C_O_* is the concentration of the component present in the feed solution, %.

#### 2.2.3. Sample Preparation

##### Sample Preparation for Spectrophotometric Analyses

A 5 mL sample (extract/juice) was placed in a 50 mL volumetric flask. Extraction was performed with methanol overnight in a refrigerator (10 °C). The methanol extracts obtained were filtered through a paper filter.

The extractions were performed in triplicate.

##### Sample Preparation for HPLC–DAD (High-Performance Liquid Chromatography with Diode-Array Detection) Analysis

After adjusting to pH 1.5 (with an HCl solution), 50 mL apple juice samples were extracted three times with ethyl acetate (50 mL). The combined extracts were filtered using anhydrous sodium sulfate, and the solvent was vacuum-evaporated. The dry residue was dissolved in 5 mL of methanol. Prior to analysis, the samples were filtered through a membrane filter (0.45 µm).

The extractions were performed in triplicate.

#### 2.2.4. Spectrophotometric Analyses

##### Determination of Total Polyphenols

The content of total polyphenols was determined by using the method of Singleton and Rossi [23], with some modifications. The methanol extract (0.1 mL) was mixed with 0.5 mL FC reagent (diluted with distilled water in a ratio of 1:4, *v*/*v*) and 1.5 mL of sodium carbonate solution (7.5%, *w*/*v*), bringing the volume of the reaction mixture to 10 mL with distilled water. The absorbance at 750 nm was measured after incubation at room temperature for 2 h. The results are presented as gallic acid equivalents (GAE) in mg per 100 mL sample (juice/extract).

##### Determination of Total Antioxidant Capacity

The total antioxidant capacity was assessed by determining the free radical-scavenging ability (DPPH test; 2,2-diphenyl-1-picrylhydrazyl) and the ferric-reducing ability power (FRAP test). Trolox, a water-soluble analogue of vitamin E, was used as a standard, and the results are expressed as equivalents of Trolox (TE) in µmol per 100 mL of sample (juice/extract).

- DPPH Test

This procedure was based on the method of Brand-Williams et al. [24], applied with the following modification: 2250 µL of a 6 × 10^−5^ M DPPH solution in methanol (prepared on the day of analysis) was mixed with 250 µL of methanol extract (diluted with distilled water in a ratio of 1:3, *v*/*v*). The absorbance at 515 nm was measured after 15 min incubation in the dark at room temperature.

- FRAP Test

This procedure was based on the method of Benzie and Strain [25], applied with some modifications. The FRAP reagent was prepared on the day of analysis by mixing 2.5 mL of a solution of TPTZ (10 mmol/L) in hydrochloric acid (40 mmol/L), 2.5 mL of an aqueous solution of FeCl_3_ (20 mmol/L), and 25 mL of acetate buffer (0.3 mol/L, pH 3.6). Then, 2250 µL of the FRAP reagent was mixed with 250 µL of the methanol extract (diluted with distilled water in a ratio of 1:3, *v*/*v*). The absorbance at 593 nm was measured after 4 min incubation in the dark at room temperature.

All spectrophotometric measurements were performed with a Helios Omega UV–Vis spectrophotometer equipped with the VISIONlite software (all from Thermo Fisher Scientific, Madison, WI, USA), using 1 cm path length cuvettes.

#### 2.2.5. HPLC–DAD Analysis

The identification and quantification of polyphenols were performed using an HPLC–DAD Agilent 1200 system, equipped with the ChemStation software (Agilent, Waldbronn, Germany). A ZORBAX Eclipse XDB-C18 (4.6 × 150 mm, 5 µm) chromatographic column (Agilent) operating at 25 °C was used in combination with an Eclipse XDB—C18 guard column (4.6 × 12.5 mm, 5µm). The detector was set to record spectra in the range of 200–600 nm.

Chromatographic separation was performed according to Schieber et al. [26]. The mobile phase consisted of 2% (*v*/*v*) aqueous acetic acid (Eluent A) and 0.5% acetic acid in water and acetonitrile (50/50, *v*/*v*) (Eluent B). The following gradient program was used: 10–55% B (50 min); 55–100% B (10 min); 100–10% B (5 min); and 10% B (5 min). The polyphenol profile at 320 nm was recorded at a flow rate of 0.4 mL/min and an injected sample volume of 10 μL. The identification of the individual components was performed by comparison with the retention time of standard substances. Standard curves were used for the quantification of the identified components.

#### 2.2.6. Determination of Total Water-Soluble Proteins

Briefly, 10 mL of chilled juice was mixed with 60 mL of chilled ethanol. After standing overnight under refrigerated conditions (4 °C), the mixture was centrifuged for 40 min at 6000× *g* min^−1^ using an MPW-251 centrifuge (MPW MED. INSTRUMENTS, Warsaw, Poland). The resulting precipitate was dissolved in 0.5 mL of distilled water.

The water-soluble protein content was determined by using the method of Bradford [27], with bovine serum albumin (BSA) as a standard.

#### 2.2.7. Statistical Analysis

The results reported in the present study are the mean values of at least three determinations. Fisher’s least significant difference test at a significance level of 0.05 was used for the comparison of the experimental values using Excel 2010 with a one-way analysis of variance (one-way ANOVA).

## 3. Results and Discussion

### 3.1. Permeate Flux

**Figure 2:**
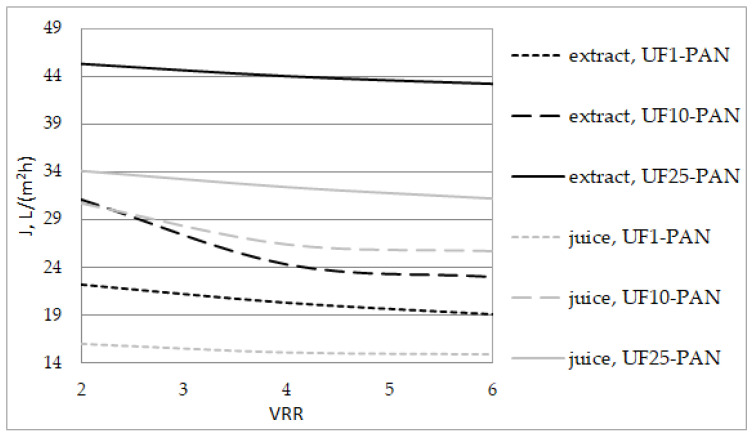
Effects of the volume reduction ratio (VRR) on the permeate flux during ultrafiltration of apple extract and juice.

Figure 2 shows the permeate flux as a function of VRR during the ultrafiltration of the apple extract and juice. For each membrane, the permeate flux decreased with the increase in the VRR. The increase in the concentration of the solution caused an increase in dynamic viscosity, which led to a reduction in the mass transfer coefficient, and thus the flux decreased. A similar trend was observed by Gaglianò et al. [4] during the nanofiltration of diafiltered apple juice. These authors considered that fouling mechanisms, such as the adsorption of particles on the membrane pore walls and pore plugging, are additional phenomena for the decrease in the permeate flux. The permeate flux of the apple extract had higher values than that of the apple juice. The increase in the molecular weight cut-off of the membrane led to an increase in the permeate flux.

### 3.2. Total Polyphenolics

#### 3.2.1. Total Polyphenolic Content

The results for the change in the total polyphenolic content during the ultrafiltration of the apple extract and juice with UF1-PAN, UF10-PAN, and UF25-PAN membranes are presented in Table 1. During ultrafiltration from VRR 2 to VRR 6, the polyphenol content increased for all the investigated membranes (*p* < 0.05). The highest values were obtained during ultrafiltration with the UF1-PAN membrane for the apple extract, and the lowest was observed when working with UF25-PAN (*p* < 0.05) for the apple juice. When the apple juice was ultrafiltered, there was no significant difference in the total polyphenols in retentates at VRR 2 between all the membranes and in retentates at VRR 4 and 6 between the UF1-PAN and UF25-PAN membranes. The UF10-PAN membrane showed the highest total polyphenols when the apple juice was subjected to ultrafiltration. The concentration of total polyphenols was higher in the retentates from the apple extract than in those from the apple juice.

#### 3.2.2. Concentration of Total Polyphenolics

**Figure 3:**
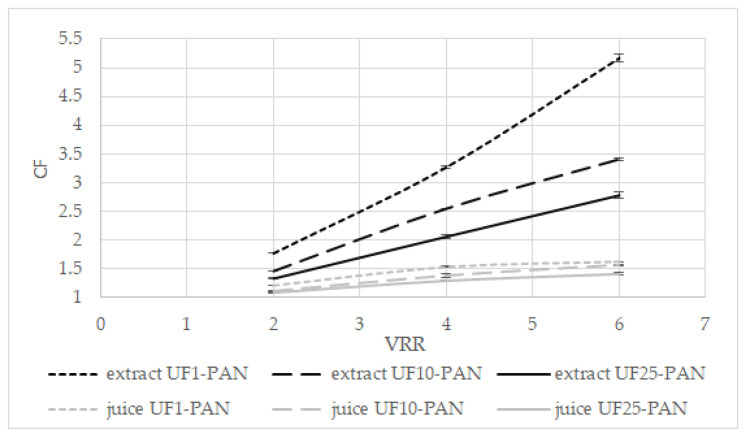
Effects of the volume reduction ratio (VRR) on the concentration factor (CF) of total polyphenols during ultrafiltration of apple extract and juice.

The effects of the volume reduction ratio (VRR) on the concentration factor (CF) of total polyphenols during the ultrafiltration of the apple extract and juice are presented in Figure 3. The concentration factor increased when the volume reduction ratio increased for all three membranes for both liquids (*p* < 0.05). When UF1-PAN was used, the concentration factor increased from 1.77 (VRR 2) to 5.17 (VRR 6) for the apple extract and from 1.21 (VRR 2) to 1.62 (VRR 6) for the apple juice. For UF25-PAN, this increase was from 1.33 (VRR 2) to 2.78 (VRR 6) for the apple extract and from 1.09 (VRR 2) to 1.41 (VRR 6) for the apple juice. It can be seen that when the molecular weight cut-off increased, the concentration factor decreased. The concentration factors were higher for the apple extract than the apple juice. It was 5.17 at VRR 6 with the UF1-PAN membrane when the apple extract was ultrafiltered, while under the same conditions for the apple juice, it was 1.62. This large difference could be explained by some matrix compounds, e.g., arabinans and galacturonic acid oligomers, that might be available in apple juice and their concentration during ultrafiltration, thus decreasing the concentration level of polyphenols. A positive correlation was found between the feed concentration and the concentration factor and recovery of polyphenolics during the ultrafiltration of the apple juice [28]. The membrane material influences also the separation and concentration of the components. Comparing the membranes with MWCO of 50 kDa from polyacrylonitrile (PAN), polyvinylidene fluoride (PVDF), and polyethersulfone (PES) for the ultrafiltration of the apple juice, it was established that the highest values of the total phenolic content were obtained when the PVDF membrane was used [29]. A higher recovery rate of antioxidant compounds was also observed, especially in terms of the total phenolic compounds, in comparison with the conventional processes in the concentrated and clarified juice obtained with NF and UF [30].

#### 3.2.3. Rejection of Total Polyphenolics

**Figure 4:**
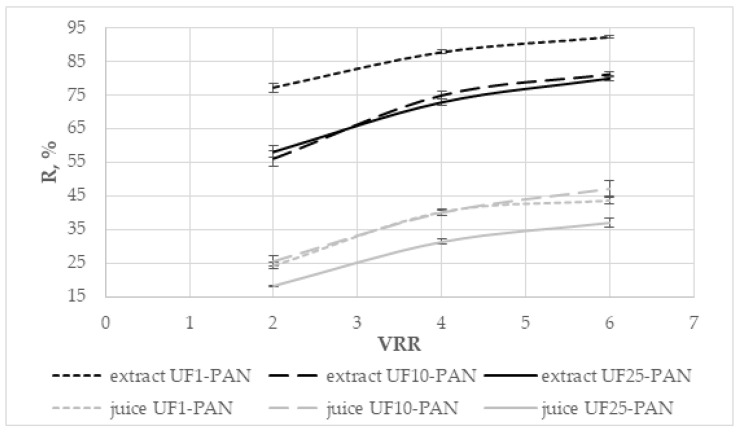
Dependence of the rejection (R, %) of total polyphenols on the volume reduction ratio (VRR) during ultrafiltration of apple extract and juice.

The dependence of the rejection (R, %) of total polyphenols on the volume reduction ratio during the ultrafiltration of the apple extract and juice is presented in Figure 4. The rejections of total polyphenols were higher for the apple extract in comparison to those of the juice. It varied from 56.04% to 92.2% for the apple extract and between 18.2% and 47.1% for the apple juice. The rejection increased with increasing the volume reduction ratio for all three membranes. Comparing the three membranes, it could be seen that the highest values of the rejection rate were obtained with the UF1-PAN membrane when the apple extract was subjected to ultrafiltration and with UF10-PAN for the apple juice. The values for the UF10-PAN and UF25-PAN membranes were comparable, and there was no significant difference between them (*p* > 0.05) for the apple extract. Huang et al. [31] established that the molecular weight cut-off of the membrane influenced the rejection rate of the juice compounds. This is probably due to the sieving effect of the membrane processes. The rejection rate of total phenolics during the ultrafiltration of Valencia orange juice was higher when a 20 kDa PS (polystyrene) membrane was used than with 30 kDa PES (polyethersulfone) [32]. The rejection rate of total phenols and flavonoids decreased when the MWCO of the membranes increased [8]. Figure 3 shows that when the apple juice was ultrafiltered, there was no significant difference between the UF10-PAN and UF1-PAN membranes (*p* > 0.05). This ultrafiltration behavior could be attributed to the complex matrix of the juice, in particular, the presence of macromolecular compounds, binding the polyphenols [21]. The retention depends on the membrane material (hydrophobic or hydrophilic) and the nature of the components [33]. 

### 3.3. Water-Soluble Proteins in Retentates Obtained via Ultrafiltration of Apple Juice

**Figure 5:**
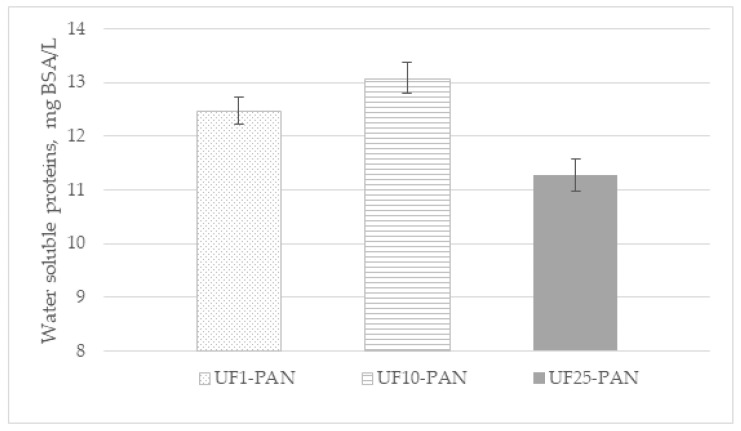
Content (mg BSA/L) of water-soluble proteins in retentates (VRR 6) obtained via ultrafiltration of apple juice.

As can be seen in Figure 5, the content of water-soluble proteins in the retentates obtained from the apple juice at VRR 6 had the highest value when using the UF10-PAN membrane and the lowest with the UF25-PAN membrane. Close values were obtained for the UF1-PAN and UF10-PAN membranes. It is known that the active protein fractions, with respect to the polyphenol-binding capacity, are in the range of 21–31 kDa in Granny Smith apple juice [34] and 12–28 kDa in Jonagold apple juice [35]. Cai et al. [29] compared the membranes with an MWCO of 50 kDa from polyacrylonitrile (PAN), polyvinylidene fluoride (PVDF), and polyethersulfone (PES) for the ultrafiltration of apple juice. The authors reported the highest values of the total protein content were observed when the PAN membrane was used.

### 3.4. HPLC Separation of Polyphenols

**Figure 6:**
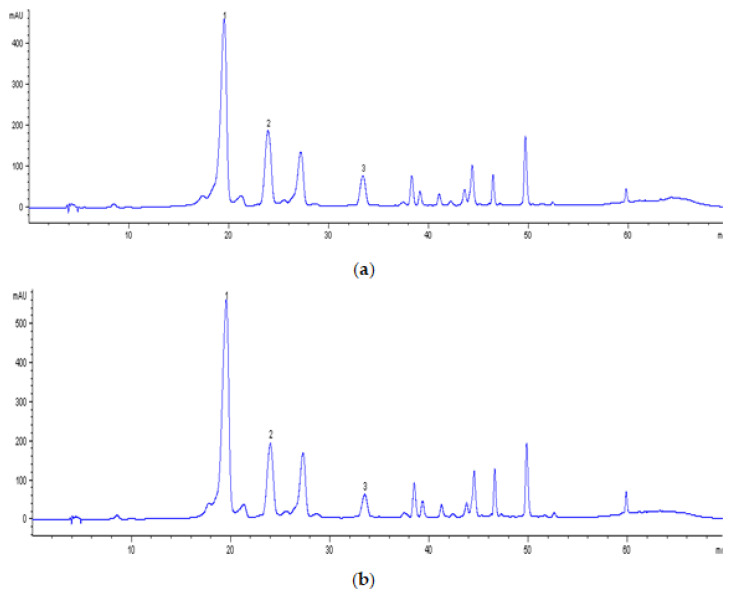
HPLC separation (320 nm) of polyphenols (1, chlorogenic acid; 2, caffeic acid; 3, *p-*coumaric acid) in apple juice subjected to ultrafiltration (UF1-PAN): (**a**) initial solution; (**b**) retentate at VRR 6.

The obtained polyphenolic profile of the apple juice, illustrated by the chromatograms in Figure 6, is consistent with that found by other authors [28,36]. It could be seen that the main compound was chlorogenic acid, the concentration of which was approximately 40% of the total polyphenolic content (based on the peak area).

### 3.5. Phenolic Acids

Analogous to the changes in the total polyphenol content, the concentration of chlorogenic acid in the retentates significantly increased for all three membranes (Table 2). These results are in agreement with those reported by Wei et al. [28], who found a significant increase in the concentration of phenolic compounds, determined by using HPLC in the retentate.

**Figure 7:**
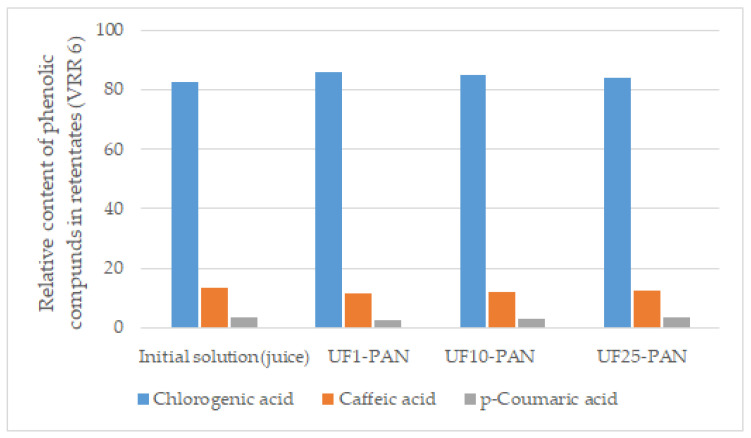
Relative content (%) of phenolic acids in the retentates (VRR 6) obtained via ultrafiltration of apple juice.

As a result of the membrane concentration, the total contents of phenolic acids increased by 15–20%. It is interesting to note that the separation process did not significantly change the ratio between the individual compounds (Figure 7). This result is important from a food quality point of view, implying that the membrane concentration of polyphenols in apple juice does not affect the product authenticity. 

### 3.6. Ferric-Reducing Antioxidant Power (FRAP)

#### 3.6.1. Changes in the Ferric-Reducing Antioxidant Power during Ultrafiltration of Apple Extract and Juice

The changes in the ferric-reducing antioxidant power (FRAP test, µmol TE/100 mL) depending on the volume reduction ratio during the ultrafiltration of the apple extract and juice are presented in Table 3. It can be seen that the ferric-reducing antioxidant power was lower in the juice than in the extract. It increased 5.46 times from the extract before ultrafiltration to the retentate at VRR 6 (UF1-PAN), and 1.66 times when the apple juice was ultrafiltered with the UF10-PAN membrane to VRR 6 (*p* < 0.05). The decrease in the permeate was 104 µmol TE/100 mL for the extract and 170.5 µmol TE/100 mL for the juice (*p* < 0.05).

#### 3.6.2. Concentration Factor of the Ferric-Reducing Antioxidant Power

**Figure 8:**
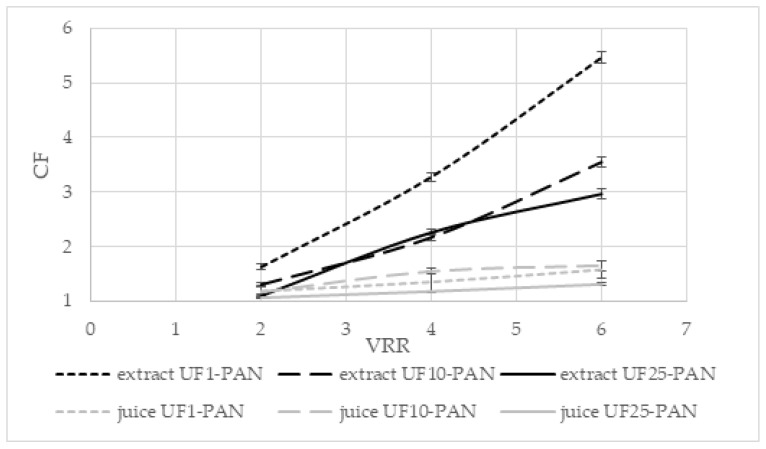
Dependence of the concentration factor (CF) of ferric-reducing antioxidant power on the volume reduction ratio (VRR) during ultrafiltration of apple extract and juice.

The dependence of the concentration factor of the ferric-reducing antioxidant power on the volume reduction ratio during the ultrafiltration of the apple extract and juice is presented in Figure 8. The concentration factors increased with increasing the volume reduction ratio for all three studied membranes. Comparing the three membranes, it could be seen that the highest values of the factor were obtained with the UF1-PAN membrane for the extract and with the UF10-PAN membrane for the juice. For the UF10-PAN and UF25-PAN membranes, close values for the extract were observed. There was no significant difference in the concentration factors at VRR 4 between UF10-PAN and UF25-PAN membranes (*p* < 0.05) for the extract and at VRR 2 between UF1-PAN and UF10-PAN membranes (*p* < 0.05) for the juice. The UF25-PAN membrane presented the lowest values of the concentration factor for both investigated liquids.

#### 3.6.3. Rejection of Ferric-Reducing Antioxidant Power

**Figure 9:**
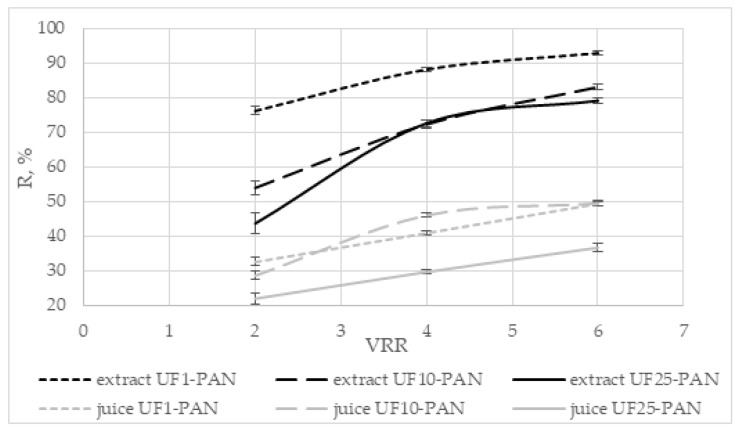
Dependence of the rejection (R, %) of ferric-reducing antioxidant power on the volume reduction ratio (VRR) during ultrafiltration of apple extract and juice.

The data for the rejection of ferric-reducing antioxidant power, shown in Figure 9, present the highest values for the UF1 membrane (apple extract) and the UF10-PAN membrane (apple juice). Close values were obtained for the UF10-PAN and UF25-PAN membranes (extract) and the UF1-PAN and UF10-PAN membranes (juice). No significant difference was observed between the rejections of UF10-PAN and UF25-PAN at VRR 4 for the extract and between UF1-PAN and UF10-PAN at VRR 6 for the juice (*p* < 0.05). During the ultrafiltration of the apple extract, the values were from 43.6% (UF25-PAN) to 92.9% (UF1-PAN) and from 21.9% (UF25-PAN) to 49.4% (UF10-PAN) for the juice. Higher values were established for the extract than for the juice. According to Cissé et al. [7], the rejection rate is related to some interactions between the solutes and the membrane material, the association of the solutes with retained macromolecules, etc. Conidi et al. [37] investigated three polymeric membranes during the ultrafiltration of blood orange juice: PS (polystyrene) at 100 kDa, PS at 50 kDa, and PAN (polyacrylonitrile) at 50 kDa. They established the highest rejection of the total antioxidant power for the 50 kDa PAN membrane and the lowest for the 100 PS kDa membrane. The rejection of the total antioxidant power during the ultrafiltration of Valencia orange juice was higher when the 20 kDa PS (polystyrene) membrane was used than with 30 kDa PES (polyethersulfone) [32].

### 3.7. Free Radical-Scavenging Ability (DPPH)

#### 3.7.1. Changes in Free Radical-Scavenging Ability

The changes in the free radical-scavenging ability (DPPH) depending on the volume reduction ratio during the ultrafiltration of the apple extract and juice are presented in Table 4. The radical-scavenging ability was positively influenced by the volume reduction ratio for both liquids (*p* < 0.05), except for the juice ultrafiltered with the UF25-PAN membrane (*p* > 0.05). Higher values were established for the extract than for the juice (*p* < 0.05). The highest values were obtained for the extract at VRR 6 with the UF1-PAN membrane (2194.4 µmol TE/100 mL).

#### 3.7.2. Concentration of the Antioxidants with Radical-Scavenging Ability

**Figure 10:**
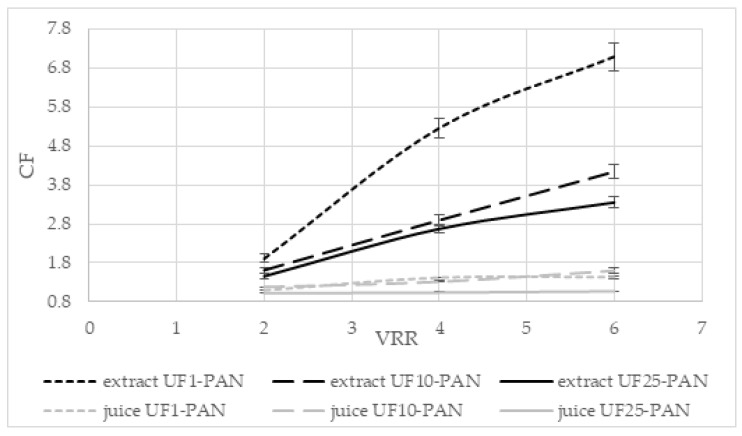
Dependence of the concentration factor (CF) of antioxidants with radical-scavenging ability on the volume reduction ratio (VRR) during ultrafiltration of apple extract and juice.

**Figure 11:**
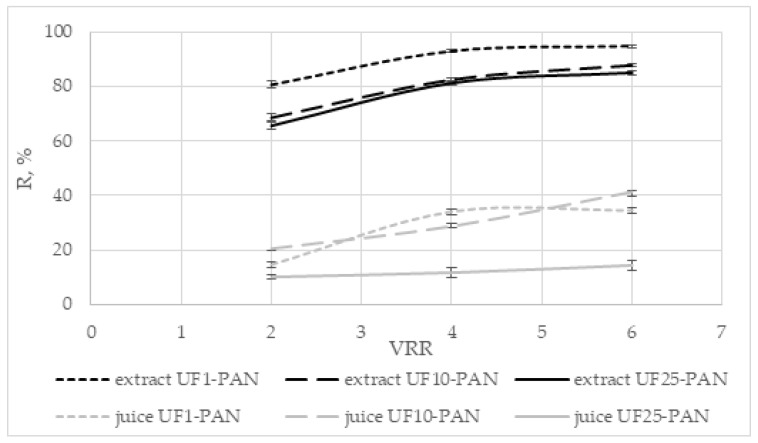
Dependence of the rejection (R, %) of antioxidants with radical-scavenging ability on the volume reduction ratio (VRR) during ultrafiltration of apple extract and juice.

The influences of the concentration factor and the rejection of the antioxidants with radical-scavenging ability on the volume reduction are presented in Figure 10 and Figure 11, respectively. The concentration factors increased with increasing the volume reduction ratio for all the investigated membranes (*p* < 0.05). Comparing the three membranes, it could be seen that the lowest values were obtained for the UF25-PAN membrane and the highest for the UF1-PAN membrane (extract), while the highest concentration factors for the juice were when UF10-PAN was used. There was no significant difference in the concentration factors at VRR 2 and VRR 4 between the UF10-PAN and UF25-PAN membranes during the ultrafiltration of the extract (*p* > 0.05).

#### 3.7.3. Rejection of the Antioxidants with Radical-Scavenging Ability

The rejections (Figure 10) were between 65.6% and 94.8% (extract) and between 10.2% and 41.4% (juice). The highest values were established with the UF1-PAN membrane (extract) and with the UF10-PAN membrane (juice). There was no significant difference in the rejection rates of all the VRRs investigated between the UF10-PAN and UF25-PAN membranes when the apple extract was ultrafiltered (*p* > 0.05). Cissé et al. [7] reported that the retention of anthocyanins decreased following a logarithmic proportion as the nominal MWCO of polyethersulphone membranes increased during the ultrafiltration of roselle extract (*Hibiscus sabdariffa* L.). A linear increase in the total anthocyanins and total ellagitannins with decreasing the nominal MWCO of polyethersulphone membranes during the ultrafiltration of blackberry (*Rubus adenotrichus* Schltdl.) juice was established by Acosta et al. [38].

## 4. Conclusions

The permeate flux, concentration, and rejection effectiveness depended on the type of liquid, the volume reduction ratio, and the molecular weight cut-off of the membranes. Chlorogenic acid was the main compound in the polyphenol profile of apple juice. The total content of phenolic acids, determined by using HPLC, increased by 15–20% as a result of the membrane concentration, and the separation process did not significantly change the ratio between the individual compounds. Ultrafiltration can be used not only for the clarification of juices but also for the concentration of polyphenolic antioxidants in order to produce apple juice with health effects.

## Figures and Tables

**Table 1:** Changes in total polyphenols (mg GAE/100 mL) during ultrafiltration of apple extract and juice.

Sample		Membranes		
		UF1-PAN	UF10-PAN	UF25-PAN
Feed solution	Extract	65.9 ± 1.9 ^a,A^	64.6 ± 1.2 ^a,A^	68.6 ± 2.1 ^a,A^
	Juice	51.8 ± 2.5 ^a,A^	60.8 ± 3 ^a,A^	58.2 ± 2.1 ^a,A^
Permeate	Extract	26.6 ± 0.8 ^b,A^	41.5 ± 1.1 ^b,B^	38.4 ± 0.9 ^b,C^
	Juice	47.5 ± 1.5 ^b,A^	50.5 ± 2 ^b,A^	51.7 ± 1.7 ^b,A^
Retentate at VRR 2	Extract	116.7 ± 3.2 ^c,A^	94.4 ± 2.1 ^c,B^	91.6 ± 1.8 ^c,B^
	Juice	62.7 ± 3 ^c,A^	67.8 ± 2.9 ^a,A^	63.2 ± 2.1 ^c,A^
Retentate at VRR 4	Extract	215.9 ± 4.1 ^d,A^	165.0 ± 3.2 ^d,B^	141.1 ± 1.9 ^d,C^
	Juice	79.4 ± 3.5 ^d,A^	84.1 ± 2.5 ^c,B^	75.2 ± 1.9 ^d,A^
Retentate at VRR 6	Extract	340.7 ± 5.0 ^e,A^	220.1 ± 2.8 ^e,B^	191.0 ± 2.4 ^e,C^
	Juice	84.2 ± 4.1 ^d,A^	95.4 ± 3.9 ^d,B^	82.0 ± 4.5 ^d,A^

Note: Different lowercase letters (a–e) show significant differences between the total polyphenols of the feed solution, permeate, and retentates (*p* < 0.05). Different upper letters (A–C) show significant differences between the polyphenol content of the samples ultrafiltered with the three membranes (*p* < 0.05).

**Table 2:** Content of phenolic acids (mg/L) in the ultrafiltration retentates (VRR 6).

Name of the Compound	Feed Solution (Apple Juice)	Membranes		
		UF1-PAN	UF10-PAN	UF25-PAN
Chlorogenic acid	35.67 ± 1.14 ^a^	44.35 ± 1.02 ^b^	43.60 ± 0.65 ^b,c^	41.53 ± 1.11 ^c^
Caffeic acid	5.78 ± 0.02 ^a^	6.00 ± 0.23 ^a^	6.21 ± 0.21 ^a^	6.26 ± 0.22 ^a^
*p*-Coumaric acid	1.61 ± 0.04 ^a^	1.28 ± 0.03 ^b^	1.61 ± 0.09 ^a^	1.72 ± 0.08 ^a^
Sum of phenolic acids	43.06	51.63	51.42	49.51

Note: Different lowercase letters (a–c) show significant differences between the content of phenolic acids of feed solution and retentates (*p* < 0.05).

**Table 3:** Changes in the ferric-reducing antioxidant power (FRAP test, µmol TE/100 mL) during ultrafiltration of apple extract and juice.

Sample		Membranes		
		UF1-PAN	UF10-PAN	UF25-PAN
Feed solution	Extract	267.6 ± 5.5 ^a,A^	279.4 ± 4.3 ^a,A^	275.3 ± 3.9 ^a,A^
	Juice	163.5 ± 6 ^a,A^	163.5 ± 6 ^a,A^	180.7 ± 8.1 ^a,A^
Permeate	Extract	104.0 ± 3.2 ^b,A^	168.1 ± 5.1 ^b,B^	170.5 ± 4.5 ^b,B^
	Juice	131.0 ± 4 ^b,A^	136.9 ± 3.7 ^b,A^	150.2 ± 6.2 ^b,A^
Retentate at VRR 2	Extract	436.3 ± 8.5 ^c,A^	364.8 ± 4.1 ^c,B^	302.5 ± 7.9 ^c,C^
	Juice	194.0 ± 5.5 ^c,A^	195.1 ± 8.5 ^c,A^	192.3 ± 4.3 ^a,A^
Retentate at VRR 4	Extract	877.3 ± 10.2 ^d,A^	606.7 ± 8.9 ^d,B^	622.4 ± 7.3 ^d,B^
	Juice	221.4 ± 7.2 ^d,A^	253.3 ± 5.9 ^d,B^	213.3 ± 8 ^d,C^
Retentate at VRR 6	Extract	1462.2 ± 12.8 ^e,A^	991.9 ± 11.0 ^e,B^	816.1 ± 13.1 ^e,C^
	Juice	258.4 ± 8 ^e,A^	270.7 ± 7.2 ^d,A^	237.0 ± 5.3 ^e,B^

Note: Different lowercase letters (a–e) show significant differences between the ferric-reducing antioxidant power of the feed solution, permeate, and retentates (*p* < 0.05). Different upper letters (A–C) show significant differences between the ferric-reducing antioxidant power of the samples ultrafiltered with the three membranes investigated (*p* < 0.05).

**Table 4:** Changes in the free radical-scavenging ability (DPPH test, µmol TE/100 mL) during ultrafiltration of apple extract and juice.

Sample		Membranes		
		UF1-PAN	UF10-PAN	UF25-PAN
Feed solution	Extract	309.6 ± 10.5 ^a,A^	360.4 ± 9.2 ^a,B^	387.4 ± 8.1 ^a,C^
	Juice	135.3 ± 4.3 ^a,A^	170.9 ± 5.2 ^a,B^	384.9 ± 10.8 ^a,C^
Permeate	Extract	114.6 ± 4.5 ^b,A^	183.3 ± 5.2 ^b,B^	194.5 ± 5.0 ^b,B^
	Juice	127.0 ± 3.8 ^b,A^	160.5 ± 5 ^b,B^	335.3 ± 5.8 ^b,C^
Retentate at VRR = 2	Extract	591.3 ± 12.4 ^c,A^	582.2 ± 10.1 ^c,A^	565.3 ± 11 ^c,A^
	Juice	148.9 ± 4.4 ^c,A^	202.1 ± 5.3 ^c,B^	393.3 ± 10.0 ^a,C^
Retentate at VRR = 4	Extract	1627.6 ± 18.5 ^d,A^	1043.3 ± 20.4 ^d,B^	1035.3 ± 15.6 ^d,B^
	Juice	192.8 ± 6.0 ^d,A^	225.3 ± 9.9 ^d,B^	400.5 ± 15.5 ^a,A^
Retentate at VRR = 6	Extract	2194.4 ± 35.1 ^e,A^	1491.7 ± 28.4 ^e,B^	1298.1 ± 32 ^e,C^
	Juice	194.2 ± 4.1 ^d,A^	273.9 ± 17.9 ^e,B^	412.9 ± 15.0 ^a,C^

Note: Different lowercase letters (a–e) show significant differences between the radical-scavenging ability of the feed solution, permeate, and retentates (*p* < 0.05). Different upper letters (A–C) show significant differences between the radical-scavenging ability of the samples ultrafiltered with the three membranes investigated (*p* < 0.05).

## Data Availability

Data is contained within the article.
